# Supplementary material for: Mapping influenza transmission in the ferret model to transmission in humans
Source: eLife. 2015 Sep 2;4:e07969. doi: 10.7554/eLife.07969 (PMC4586390; doi:10.7554/eLife.07969)
Supplement: Figure 1—source data 1. — DOI: http://dx.doi.org/10.7554/eLife.07969.004 [file elife07969s001.docx]

**Figure 1 – source data 1:** Estimates of human household SAR.

| **Subtype** | **Location** | **Year** | **SAR estimate** | **Reference** |
| --- | --- | --- | --- | --- |
| **H1N1** | Hong Kong, China | 2009 | 0.06^*^ | (Klick et al. 2011) |
|  | Hong Kong, China | 2009 | 0.07^†^ | (Klick et al. 2011) |
|  | Germany | 2007-11 | 0.375 | (Suess et al. 2012a) |
|  | Germany | 2007-8 | 0.262 | (Buchholz et al. 2010) |
|  | Seattle, WA | 1978-9 | 0.306 | (Longini et al. 1982) |
|  | Seattle, WA | 1978-9 | 0.44 | (FOX et al. 1982) |
|  | Cirencester, England | 1951 | 0.356 | (Longini and Koopman 1982) |
| **H3N2** | Ann Arbor, MI | 2010-11 | 0.153 | (Petrie et al. 2013) |
|  | Ulaanbaatar City, Mongolia | 2010-11 | 0.057 | (Nukiwa-Souma et al. 2012) |
|  | Hong Kong, China | 2009 | 0.07^*^ | (Klick et al. 2011) |
|  | Hong Kong, China | 2009 | 0.09^†^ | (Klick et al. 2011) |
|  | Hong Kong, China | 2009 | 0.09 | (Cowling et al. 2010) |
|  | France | 2008-9 | 0.158 | (Canini et al. 2010) |
|  | Germany | 2007-11 | 0.478 | (Suess et al. 2012a) |
|  | France | 2000 | 0.383 | (Carrat et al. 2002) |
|  | France | 1999-2000 | 0.241 | (Viboud et al. 2004) |
|  | France | 1999-2000 | 0.260 | (Cauchemez et al. 2004) |
|  | Tecumseh, MI | 1985 | 0.143 | (Longini and Monto 1988) |
|  | Tecumseh, MI | 1977-8; 1980-1 | 0.260 | (Longini et al. 1988) |
|  | Tecumseh, MI | 1978-9 | 0.147 | (Longini et al. 1982) |
|  | Seattle, WA | 1977-9 | 0.206 | (Longini et al. 1982) |
|  | Seattle, WA | 1975-8 | 0.53 | (FOX et al. 1982) |
|  | Port Chalmers, New Zealand | 1973-1975 | 0.547 | (Jennings and Miles 1978) |
| **pH1N1** | Ann Arbor, MI | 2010-11 | 0.029 | (Petrie et al. 2013) |
|  | Berlin, Germany | 2009-11 | 0.23 | (Suess et al. 2012b) |
|  | Kenya | 2009-10 | 0.060 | (Kim et al. 2012) |
|  | Thailand | 2009-10 | 0.472 | (Khuntirat et al. 2014) |
|  | Bangkok, Thailand | 2009 | 0.19 | (Simmerman et al. 2011) |
|  | Hong Kong, China | 2009 | 0.15^*^ | (Klick et al. 2011) |
|  | Hong Kong, China | 2009 | 0.07^†^ | (Klick et al. 2011) |
|  | Hong Kong, China | 2009 | 0.08 | (Cowling et al. 2010) |
|  | Hong Kong, China | 2009 | 0.059 | (Leung et al. 2011) |
|  | United States | 2009 | 0.273 | (Yang et al. 2009) |
|  | United States | 2009 | 0.1 | (Cauchemez et al. 2009) |
|  | Victoria, Australia | 2009 | 0.148 | (Gemert et al. 2011) |
|  | Victoria, Australia | 2009 | 0.33 | (Looker et al. 2010) |
|  | Vietnam | 2009 | 0.186 | (Thai et al. 2014) |
|  | Washington, United States | 2009 | 0.06 | (Sugimoto et al. 2011) |
|  | Germany | 2009 | 0.26 | (Suess et al. 2010) |
|  | Germany | 2009 | 0.101 | (Remschmidt et al. 2013) |
|  | Edmonton, Canada | 2009 | 0.302 | (Sikora et al. 2010) |
|  | Ontario, Canada | 2009 | 0.103 | (Savage et al. 2011) |
|  | Los Lagos, Chile | 2009 | 0.35 | (Pedroni et al. 2010) |
|  | United Kingdom | 2009 | 0.105 | (Pebody et al. 2011) |
|  | Quebec City, Canada | 2009 | 0.29 | (Papenburg et al. 2010) |
|  | Kobe City, Japan | 2009 | 0.076 | (Odaira et al. 2009) |
|  | Japan | 2009 | 0.114 | (Nishiura and Oshitani 2011) |
|  | Japan | 2009 | 0.079 | (Hirotsu et al. 2011) |
|  | Osaka, Japan | 2009 | 0.261 | (Komiya et al. 2010) |
|  | San Antonio, TX | 2009 | 0.148 | (Morgan et al. 2010) |
|  | Riyadh, Saudi Arabia | 2009 | 0.169 | (Mohamed et al. 2012) |
|  | San Antonio, TX | 2009 | 0.037 | (Loustalot et al. 2011) |
|  | Hangzhou, China | 2009 | 0.087 | (Liu et al. 2010) |
|  | Seoul, South Korea | 2009 | 0.279 | (Lee et al. 2010) |
|  | British Columbia, Canada | 2009 | 0.22 | (Janjua et al. 2012) |
|  | New York, NY | 2009 | 0.19 | (Jackson et al. 2011) |
|  | New York, NY | 2009 | 0.113 | (France et al. 2010) |
|  | Florida, United States | 2009 | 0.143 | (Doyle and Hopkins 2011) |
|  | Taiwan | 2009 | 0.27 | (Chang et al. 2011) |
|  | Kenya | 2009 | 0.26 | (Tabu et al. 2009) |
|  | Western Australia | 2009 | 0.15 | (Carcione et al. 2010) |
|  | Western Australia | 2009 | 0.145 | (Carcione et al. 2011) |
|  | Yazd, Iran | 2009 | 0.139 | (Behnaz et al. 2012) |
|  | South Africa | 2009 | 0.17 | (Archer et al. 2012) |
|  | Germany | 2007-11 | 0.154 | (Suess et al. 2012a) |
| **H2N2** | Tokyo, Japan | 1957 | 0.0706 | (Nishiura and Chowell 2007) |
|  | Osaka, Japan | 1957 | 0.0907 | (Nishiura and Chowell 2007) |
|  | Kansas City, MO | 1957 | 0.142 | (Chin et al. 1960) |
| **H7N9** | China | 2013-14 | 0.014 | (Yang et al. 2015) |
|  | China | 2013 | 0.129 | (Li et al. 2014) |
|  | Shanghai, China | 2013 | 0.20 | (Hu et al. 2014) |
|  | Shaoxing City, China | 2013 | 0 | (Chen et al. 2013) |
| **H5N1** | Cambodia | 2011 | 0 | (Chea et al. 2014) |
|  | Indonesia | 2006 | 0.29 | (Yang et al. 2007) |
|  | Indonesia | 2005-9 | 0.055 | (Aditama et al. 2012) |
|  | Hong Kong, China | 1997 | 0.048 | (Katz et al. 1999) |
| **H7N2** | Wales | 2007 | 0.111 | (Eames et al. 2010) |
|  | New York, NY | 2003 | 0 | (Ostrowsky et al. 2012) |
| **H7N7** | Netherlands | 2003 | 0.097 | (Du Ry van Beest Holle et al. 2005) |
|  | Netherlands | 2003 | 0.011 | (Koopmans et al. 2004) |
| **H9N2** | Hong Kong, China | 1999 | 0 | (Uyeki et al. 2002) |
| **H7N3** | United Kingdom | 2006 | 0 | (Nguyen-Van-Tam et al. 2006) |
|  | British Columbia, Canada | 2004 | 0 | (Tweed et al. 2004) |

^*^Estimate is for children only

^†^Estimate is for adults only

**References**

Aditama, T. Y., G. Samaan, R. Kusriastuti, O. D. Sampurno, W. Purba, Misriyah, H. Santoso, A. Bratasena, A. Maruf, E. Sariwati, V. Setiawaty, K. Glass, K. Lokuge, P. M. Kelly, and I. N. Kandun. 2012. Avian influenza H5N1 transmission in households, Indonesia. PloS one 7:e29971.

Archer, B. N., G. A. Timothy, C. Cohen, S. Tempia, M. Huma, L. Blumberg, D. Naidoo, A. Cengimbo, and B. D. Schoub. 2012. Introduction of 2009 pandemic influenza A virus subtype H1N1 into South Africa: clinical presentation, epidemiology, and transmissibility of the first 100 cases. The Journal of infectious diseases 206 Suppl :S148–53.

Behnaz, F., M. Mohammadzadeh, and M. Sadeghian. 2012. Household transmission of 2009 H1N1 influenza virus in Yazd, Iran. Journal of infection and public health 5:275–80.

Buchholz, U., S. Brockmann, S. Duwe, B. Schweiger, M. an der Heiden, B. Reinhardt, and S. Buda. 2010. Household transmissibility and other characteristics of seasonal oseltamivir-resistant influenza A(H1N1) viruses, Germany, 2007-8. Euro Surveillance 15.

Canini, L., L. Andréoletti, P. Ferrari, R. D’Angelo, T. Blanchon, M. Lemaitre, L. Filleul, J.-P. Ferry, M. Desmaizieres, S. Smadja, A.-J. Valleron, and F. Carrat. 2010. Surgical mask to prevent influenza transmission in households: a cluster randomized trial. PloS one 5:e13998.

Carcione, D., C. Giele, L. S. Goggin, K. S. Kwan, D. W. Smith, G. K. Dowse, D. B. Mak, and P. Effler. 2010. Association between 2009 seasonal influenza vaccine and influenza-like illness during the 2009 pandemic: preliminary results of a large household transmission study in Western Australia. Euro surveillance : bulletin Européen sur les maladies transmissibles = European communicable disease bulletin 15.

Carcione, D., C. M. Giele, L. S. Goggin, K. S. Kwan, D. W. Smith, G. K. Dowse, D. B. Mak, and P. Effler. 2011. Secondary attack rate of pandemic influenza A(H1N1) 2009 in Western Australian households, 29 May-7 August 2009. Euro surveillance : bulletin Européen sur les maladies transmissibles = European communicable disease bulletin 16.

Carrat, F., C. Sahler, S. Rogez, M. Leruez-Ville, F. Freymuth, C. Le Gales, M. Bungener, B. Housset, M. Nicolas, and C. Rouzioux. 2002. Influenza burden of illness: estimates from a national prospective survey of household contacts in France. Archives of internal medicine 162:1842–8.

Cauchemez, S., F. Carrat, C. Viboud, A. J. Valleron, and P. Y. Boëlle. 2004. A Bayesian MCMC approach to study transmission of influenza: application to household longitudinal data. Statistics in medicine 23:3469–87.

Cauchemez, S., C. A. Donnelly, C. Reed, A. C. Ghani, C. Fraser, C. K. Kent, L. Finelli, and N. M. Ferguson. 2009. Household transmission of 2009 pandemic influenza A (H1N1) virus in the United States. The New England journal of medicine 361:2619–27.

Chang, L.-Y., W.-H. Chen, C.-Y. Lu, P.-L. Shao, T.-Y. Fan, A.-L. Cheng, and L.-M. Huang. 2011. Household transmission of Pandemic (H1N1) 2009 Virus, Taiwan. Emerging infectious diseases 17:1928–31.

Chea, N., S. D. Yi, S. Rith, H. Seng, V. Ieng, C. Penh, S. Mardy, D. Laurent, B. Richner, T. Sok, S. Ly, P. Kitsutani, N. Asgari, M. C. Roces, P. Buchy, and A. Tarantola. 2014. Two clustered cases of confirmed influenza A(H5N1) virus infection, Cambodia, 2011. Euro surveillance : bulletin Européen sur les maladies transmissibles = European communicable disease bulletin 19.

Chen, E., Y. Chen, L. Fu, Z. Chen, Z. Gong, H. Mao, D. Wang, M. Y. Ni, P. Wu, Z. Yu, T. He, Z. Li, J. Gao, S. Liu, Y. Shu, B. J. Cowling, S. Xia, and H. Yu. 2013. Human infection with avian influenza A(H7N9) virus re-emerges in China in winter 2013. Euro surveillance : bulletin Européen sur les maladies transmissibles = European communicable disease bulletin 18.

Chin, T. D. Y., J. F. Foley, I. L. Doto, C. R. Gravelle, and J. Weston. 1960. Morbidity and mortality characteristics of Asian strain influenza. Public health reports 75:149–158.

Cowling, B. J., K. H. Chan, V. J. Fang, L. L. H. Lau, H. C. So, R. O. P. Fung, E. S. K. Ma, A. S. K. Kwong, C.-W. Chan, W. W. S. Tsui, H.-Y. Ngai, D. W. S. Chu, P. W. Y. Lee, M.-C. Chiu, G. M. Leung, and J. S. M. Peiris. 2010. Comparative Epidemiology of Pandemic and Seasonal Influenza A in Households. New England Journal of Medicine 362:2175–2184.

Doyle, T. J., and R. S. Hopkins. 2011. Low secondary transmission of 2009 pandemic influenza A (H1N1) in households following an outbreak at a summer camp: relationship to timing of exposure. Epidemiology and infection 139:45–51.

Eames, K. T. D., C. Webb, K. Thomas, J. Smith, R. Salmon, and J. M. F. Temple. 2010. Assessing the role of contact tracing in a suspected H7N2 influenza A outbreak in humans in Wales. BMC infectious diseases 10:141.

FOX, J. P., M. K. COONEY, C. E. HALL, and H. M. FOY. 1982. INFLUENZAVIRUS INFECTIONS IN SEATTLE FAMILIES, 1975-1979: II. PATTERN OF INFECTION IN INVADED HOUSEHOLDS AND RELATION OF AGE AND PRIOR ANTIBODY TO OCCURRENCE OF INFECTION AND RELATED ILLNESS. Am. J. Epidemiol. 116:228–242.

France, A. M., M. Jackson, S. Schrag, M. Lynch, C. Zimmerman, M. Biggerstaff, and J. Hadler. 2010. Household transmission of 2009 influenza A (H1N1) virus after a school-based outbreak in New York City, April-May 2009. The Journal of infectious diseases 201:984–92.

Gemert, C. van, M. Hellard, E. S. McBryde, J. Fielding, T. Spelman, N. Higgins, R. Lester, H. Vally, and I. Bergeri. 2011. Intrahousehold Transmission of Pandemic (H1N1) 2009 Virus, Victoria, Australia. Emerging Infectious Disease journal 17:1599.

Hirotsu, N., T. Hasegawa, H. Ikematsu, N. Iwaki, and N. Kawai. 2011. Transmission of pandemic H1N1 2009 influenza in households and school settings : comparison with seasonal influenza. Infl 5:324–327.

Hu, J., Y. Zhu, B. Zhao, J. Li, L. Liu, K. Gu, W. Zhang, H. Su, Z. Teng, S. Tang, Z. Yuan, Z. Feng, and F. Wu. 2014. Limited human-to-human transmission of avian influenza A(H7N9) virus, Shanghai, China, March to April 2013. Euro surveillance : bulletin Européen sur les maladies transmissibles = European communicable disease bulletin 19.

Jackson, M. L., A. M. France, K. Hancock, X. Lu, V. Veguilla, H. Sun, F. Liu, J. Hadler, B. H. Harcourt, D. H. Esposito, C. M. Zimmerman, J. M. Katz, A. M. Fry, and S. J. Schrag. 2011. Serologically confirmed household transmission of 2009 pandemic influenza A (H1N1) virus during the first pandemic wave--New York City, April-May 2009. Clinical infectious diseases : an official publication of the Infectious Diseases Society of America 53:455–62.

Janjua, N. Z., D. M. Skowronski, T. S. Hottes, W. Osei, E. Adams, M. Petric, M. Lem, P. Tang, G. De Serres, D. M. Patrick, and D. Bowering. 2012. Transmission dynamics and risk factors for pandemic H1N1-related illness: outbreak investigation in a rural community of British Columbia, Canada. Influenza and other respiratory viruses 6:e54–62.

Jennings, L. C., and J. A. Miles. 1978. A study of acute respiratory disease in the community of Port Chalmers. II. Influenza A/Port Chalmers/1/73: intrafamilial spread and the effect of antibodies to the surface antigens. The Journal of hygiene 81:67–75.

Katz, J. M., W. Lim, C. B. Bridges, T. Rowe, J. Hu-Primmer, X. Lu, R. A. Abernathy, M. Clarke, L. Conn, H. Kwong, M. Lee, G. Au, Y. Y. Ho, K. H. Mak, N. J. Cox, and K. Fukuda. 1999. Antibody response in individuals infected with avian influenza A (H5N1) viruses and detection of anti-H5 antibody among household and social contacts. The Journal of infectious diseases 180:1763–70.

Khuntirat, B., I.-K. Yoon, M. Chittaganpitch, W. S. Krueger, K. Supawat, P. J. Blair, S. D. Putnam, R. V Gibbons, D. Buddhari, P. Sawanpanyalert, G. L. Heil, J. A. Friary, and G. C. Gray. 2014. High rate of A(H1N1)pdm09 infections among rural Thai villagers, 2009-2010. PloS one 9:e106751.

Kim, C. Y., R. F. Breiman, L. Cosmas, A. Audi, B. Aura, G. Bigogo, H. Njuguna, E. Lebo, L. Waiboci, M. K. Njenga, D. R. Feikin, and M. A. Katz. 2012. Secondary household transmission of 2009 pandemic influenza A (H1N1) virus among an urban and rural population in Kenya, 2009-2010. PloS one 7:e38166.

Klick, B., H. Nishiura, S. Ng, V. J. Fang, G. M. Leung, J. S. M. Peiris, and B. J. Cowling. 2011. Transmissibility of seasonal and pandemic influenza in a cohort of households in Hong Kong in 2009. Epidemiology (Cambridge, Mass.) 22:793–6.

Komiya, N., Y. Gu, H. Kamiya, Y. Yahata, Y. Yasui, K. Taniguchi, and N. Okabe. 2010. Household transmission of pandemic 2009 influenza A (H1N1) virus in Osaka, Japan in May 2009. The Journal of infection 61:284–8.

Koopmans, M., B. Wilbrink, M. Conyn, G. Natrop, H. van der Nat, H. Vennema, A. Meijer, J. van Steenbergen, R. Fouchier, A. Osterhaus, and A. Bosman. 2004. Transmission of H7N7 avian influenza A virus to human beings during a large outbreak in commercial poultry farms in the Netherlands. Lancet (London, England) 363:587–93.

Lee, D. H., C. W. Kim, J.-H. Kim, J. S. Lee, M. K. Lee, J. C. Choi, B. W. Choi, S.-H. Choi, and J.-W. Chung. 2010. Risk factors for laboratory-confirmed household transmission of pandemic H1N1 2009 infection. American journal of infection control 38:e43–5.

Leung, Y. H., M. P. Li, and S. K. Chuang. 2011. A school outbreak of pandemic (H1N1) 2009 infection: assessment of secondary household transmission and the protective role of oseltamivir. Epidemiology and infection 139:41–4.

Li, Q., L. Zhou, M. Zhou, Z. Chen, F. Li, H. Wu, N. Xiang, E. Chen, F. Tang, D. Wang, L. Meng, Z. Hong, W. Tu, Y. Cao, L. Li, F. Ding, B. Liu, M. Wang, R. Xie, R. Gao, X. Li, T. Bai, S. Zou, J. He, J. Hu, Y. Xu, C. Chai, S. Wang, Y. Gao, L. Jin, Y. Zhang, H. Luo, H. Yu, J. He, Q. Li, X. Wang, L. Gao, X. Pang, G. Liu, Y. Yan, H. Yuan, Y. Shu, W. Yang, Y. Wang, F. Wu, T. M. Uyeki, and Z. Feng. 2014. Epidemiology of human infections with avian influenza A(H7N9) virus in China. The New England journal of medicine 370:520–32.

Liu, S.-L., Z.-R. Zhang, C. Wang, Y. Dong, L.-B. Cui, X.-H. Yang, Z. Sun, J. Wang, J. Chen, R.-J. Huang, F. Miao, B. Ruan, L. Xie, H.-X. He, and J. Deng. 2010. 2009 pandemic characteristics and controlling experiences of influenza H1N1 virus 1 year after the inception in Hangzhou, China. Journal of medical virology 82:1985–95.

Longini, I. M., and J. S. Koopman. 1982. Household and community transmission parameters from final distributions of infections in households. http://www.jstor.org/stable/2530294.

Longini, I. M., J. S. Koopman, M. Haber, and G. A. Cotsonis. 1988. Statistical inference for infectious diseases. Risk-specific household and community transmission parameters. American journal of epidemiology 128:845–59.

Longini, I. M., J. S. Koopman, A. S. Monto, and J. P. Fox. 1982. Estimating household and community transmission parameters for influenza. Am. J. Epidemiol. 115:736–751.

Longini, I. M., and A. S. Monto. 1988. Efficacy of virucidal nasal tissues in interrupting familial transmission of respiratory agents. A field trial in Tecumseh, Michigan. American journal of epidemiology 128:639–44.

Looker, C., K. Carville, K. Grant, and H. Kelly. 2010. Influenza A (H1N1) in Victoria, Australia: a community case series and analysis of household transmission. PloS one 5:e13702.

Loustalot, F., B. J. Silk, A. Gaither, T. Shim, M. Lamias, F. Dawood, O. W. Morgan, D. Fishbein, S. Guerra, J. R. Verani, S. A. Carlson, V. P. Fonseca, and S. J. Olsen. 2011. Household transmission of 2009 pandemic influenza A (H1N1) and nonpharmaceutical interventions among households of high school students in San Antonio, Texas. Clinical infectious diseases : an official publication of the Infectious Diseases Society of America 52 Suppl 1:S146–53.

Mohamed, A. G., A. A. BinSaeed, H. Al-Habib, and H. Al-Saif. 2012. Communicability of H1N1 and seasonal influenza among household contacts of cases in large families. Influenza and other respiratory viruses 6:e25–9.

Morgan, O. W., S. Parks, T. Shim, P. A. Blevins, P. M. Lucas, R. Sanchez, N. Walea, F. Loustalot, M. R. Duffy, M. J. Shim, S. Guerra, F. Guerra, G. Mills, J. Verani, B. Alsip, S. Lindstrom, B. Shu, S. Emery, A. L. Cohen, M. Menon, A. M. Fry, F. Dawood, V. P. Fonseca, and S. J. Olsen. 2010. Household transmission of pandemic (H1N1) 2009, San Antonio, Texas, USA, April-May 2009. Emerging infectious diseases 16:631–7.

Nguyen-Van-Tam, J. S., P. Nair, P. Acheson, A. Baker, M. Barker, S. Bracebridge, J. Croft, J. Ellis, R. Gelletlie, N. Gent, S. Ibbotson, C. Joseph, H. Mahgoub, P. Monk, T. W. Reghitt, T. Sundkvist, C. Sellwood, J. Simpson, J. Smith, J. M. Watson, M. Zambon, and N. Lightfoot. 2006. Outbreak of low pathogenicity H7N3 avian influenza in UK, including associated case of human conjunctivitis. Euro surveillance : bulletin Européen sur les maladies transmissibles = European communicable disease bulletin 11:E060504.2.

Nishiura, H., and G. Chowell. 2007. Household and community transmission of the Asian influenza A (H2N2) and influenza B viruses in 1957 and 1961. The Southeast Asian journal of tropical medicine and public health 38:1075–83.

Nishiura, H., and H. Oshitani. 2011. Household transmission of influenza (H1N1-2009) in Japan: age-specificity and reduction of household transmission risk by zanamivir treatment. The Journal of international medical research 39:619–28.

Nukiwa-Souma, N., A. Burmaa, T. Kamigaki, I. Od, N. Bayasgalan, B. Darmaa, A. Suzuki, P. Nymadawa, and H. Oshitani. 2012. Influenza transmission in a community during a seasonal influenza A(H3N2) outbreak (2010-2011) in Mongolia: a community-based prospective cohort study. PloS one 7:e33046.

Odaira, F., H. Takahashi, T. Toyokawa, Y. Tsuchihashi, T. Kodama, Y. Yahata, T. Sunagawa, K. Taniguchi, and N. Okabe. 2009. Assessment of secondary attack rate and effectiveness of antiviral prophylaxis among household contacts in an influenza A(H1N1)v outbreak in Kobe, Japan, May-June 2009. Euro surveillance : bulletin Européen sur les maladies transmissibles = European communicable disease bulletin 14.

Ostrowsky, B., A. Huang, W. Terry, D. Anton, B. Brunagel, L. Traynor, S. Abid, G. Johnson, M. Kacica, J. Katz, L. Edwards, S. Lindstrom, A. Klimov, and T. M. Uyeki. 2012. Low pathogenic avian influenza A (H7N2) virus infection in immunocompromised adult, New York, USA, 2003. Emerging infectious diseases 18:1128–31.

Papenburg, J., M. Baz, M.-È. Hamelin, C. Rhéaume, J. Carbonneau, M. Ouakki, I. Rouleau, I. Hardy, D. Skowronski, M. Roger, H. Charest, G. De Serres, and G. Boivin. 2010. Household transmission of the 2009 pandemic A/H1N1 influenza virus: elevated laboratory‐confirmed secondary attack rates and evidence of asymptomatic infections. Clinical infectious diseases : an official publication of the Infectious Diseases Society of America 51:1033–41.

Pebody, R. G., R. Harris, G. Kafatos, M. Chamberland, C. Campbell, J. S. Nguyen-Van-Tam, E. McLean, N. Andrews, P. J. White, E. Wynne-Evans, J. Green, J. Ellis, T. Wreghitt, S. Bracebridge, C. Ihekweazu, I. Oliver, G. Smith, C. Hawkins, R. Salmon, B. Smyth, J. McMenamin, M. Zambon, N. Phin, and J. M. Watson. 2011. Use of antiviral drugs to reduce household transmission of pandemic (H1N1) 2009, United Kingdom. Emerging infectious diseases 17:990–9.

Pedroni, E., M. Garcia, V. Espinola, A. Guerrero, C. Gonzalez, A. Olea, M. Calvo, B. Martorell, M. Winkler, M. Carrasco, J. Vergara, J. Ulloa, A. Carrazana, O. Mujica, J. Villarroel, M. Labrana, M. Vargas, P. Gonzalez, L. Caceres, C. Zamorano, R. Momberg, G. Munoz, J. Rocco, V. Bosque, A. Gallardo, J. Elgueta, and J. Vega. 2010. Outbreak of 2009 pandemic influenza A(H1N1), Los Lagos, Chile, April-June 2009. Euro surveillance : bulletin Européen sur les maladies transmissibles = European communicable disease bulletin 15.

Petrie, J. G., S. E. Ohmit, B. J. Cowling, E. Johnson, R. T. Cross, R. E. Malosh, M. G. Thompson, and A. S. Monto. 2013. Influenza transmission in a cohort of households with children: 2010-2011. PloS one 8:e75339.

Remschmidt, C., P. Stöcker, M. an der Heiden, T. Suess, M. Luchtenberg, S. B. Schink, B. Schweiger, W. Haas, and U. Buchholz. 2013. Preventable and non-preventable risk factors for influenza transmission and hygiene behavior in German influenza households, pandemic season (H1N1) 2009/2010. Influenza and other respiratory viruses 7:418–25.

Du Ry van Beest Holle, M., A. Meijer, M. Koopmans, and C. M. de Jager. 2005. Human-to-human transmission of avian influenza A/H7N7, The Netherlands, 2003. Euro surveillance : bulletin Européen sur les maladies transmissibles = European communicable disease bulletin 10:264–8.

Savage, R., M. Whelan, I. Johnson, E. Rea, M. LaFreniere, L. C. Rosella, F. Lam, T. Badiani, A.-L. Winter, D. J. Carr, C. Frenette, M. Horn, K. Dooling, M. Varia, A.-M. Holt, V. Sunil, C. Grift, E. Paget, M. King, J. Barbaro, and N. S. Crowcroft. 2011. Assessing secondary attack rates among household contacts at the beginning of the influenza A (H1N1) pandemic in Ontario, Canada, April-June 2009: a prospective, observational study. BMC public health 11:234.

Sikora, C., S. Fan, R. Golonka, D. Sturtevant, J. Gratrix, B. E. Lee, J. Jaipaul, and M. Johnson. 2010. Transmission of pandemic influenza A (H1N1) 2009 within households: Edmonton, Canada. Journal of clinical virology : the official publication of the Pan American Society for Clinical Virology 49:90–3.

Simmerman, J. M., P. Suntarattiwong, J. Levy, R. G. Jarman, S. Kaewchana, R. V Gibbons, B. J. Cowling, W. Sanasuttipun, S. A. Maloney, T. M. Uyeki, L. Kamimoto, and T. Chotipitayasunondh. 2011. Findings from a household randomized controlled trial of hand washing and face masks to reduce influenza transmission in Bangkok, Thailand. Influenza and other respiratory viruses 5:256–67.

Suess, T., U. Buchholz, S. Dupke, R. Grunow, M. an der Heiden, A. Heider, B. Biere, B. Schweiger, W. Haas, and G. Krause. 2010. Shedding and transmission of novel influenza virus A/H1N1 infection in households--Germany, 2009. American journal of epidemiology 171:1157–64.

Suess, T., C. Remschmidt, S. B. Schink, B. Schweiger, A. Heider, J. Milde, A. Nitsche, K. Schroeder, J. Doellinger, C. Braun, W. Haas, G. Krause, and U. Buchholz. 2012a. Comparison of shedding characteristics of seasonal influenza virus (sub)types and influenza A(H1N1)pdm09; Germany, 2007-2011. PloS one 7:e51653.

Suess, T., C. Remschmidt, S. B. Schink, B. Schweiger, A. Nitsche, K. Schroeder, J. Doellinger, J. Milde, W. Haas, I. Koehler, G. Krause, and U. Buchholz. 2012b. The role of facemasks and hand hygiene in the prevention of influenza transmission in households: results from a cluster randomised trial; Berlin, Germany, 2009-2011. BMC infectious diseases 12:26.

Sugimoto, J. D., N. N. Borse, M. L. Ta, L. J. Stockman, G. E. Fischer, Y. Yang, M. E. Halloran, I. M. Longini, and J. S. Duchin. 2011. The Effect of Age on Transmission of 2009 Pandemic Influenza A (H1N1) in a Camp and Associated Households. Epidemiology (Cambridge, Mass.) 22:10.1097/EDE.0b013e3182060ca5.

Tabu, C., S. Sharif, P. Okoth, J. Kioko, C. Nzioka, P. Muthoka, M. Ope, S. Makama, R. Kalani, W. Ochieng, and others. 2009. Introduction and transmission of 2009 pandemic influenza A (H1N1) Virus-Kenya, June-July 2009. Morbidity and Mortality Weekly Report 58:1143–1146.

Thai, P. Q., L. Q. Mai, M. R. A. Welkers, N. L. K. Hang, L. T. Thanh, V. T. V. Dung, N. T. T. Yen, T. N. Duong, L. N. M. Hoa, D. D. Thoang, H. T. H. Trang, M. D. de Jong, H. Wertheim, N. T. Hien, P. Horby, and A. Fox. 2014. Pandemic H1N1 virus transmission and shedding dynamics in index case households of a prospective Vietnamese cohort. The Journal of infection 68:581–90.

Tweed, S. A., D. M. Skowronski, S. T. David, A. Larder, M. Petric, W. Lees, Y. Li, J. M. Katz, M. Krajden, R. Tellier, C. Halpert, M. Hirst, C. Astell, D. Lawrence, and A. Mak. 2004. Human Illness from Avian Influenza H7N3, British Columbia. Emerging Infectious Disease journal 10:2196.

Uyeki, T. M., Y.-H. Chong, J. M. Katz, W. Lim, Y.-Y. Ho, S. S. Wang, T. H. F. Tsang, W. W.-Y. Au, S.-C. Chan, T. Rowe, J. Hu-Primmer, J. C. Bell, W. W. Thompson, C. B. Bridges, N. J. Cox, K.-H. Mak, and K. Fukuda. 2002. Lack of Evidence for Human-to-Human Transmission of Avian Influenza A (H9N2) Viruses in Hong Kong, China 1999. Emerging Infectious Diseases 8:154–159.

Viboud, C., P.-Y. Boëlle, S. Cauchemez, A. Lavenu, A.-J. Valleron, A. Flahault, and F. Carrat. 2004. Risk factors of influenza transmission in households. The British journal of general practice : the journal of the Royal College of General Practitioners 54:684–9.

Yang, Y., M. E. Halloran, J. D. Sugimoto, and I. M. Longini. 2007. Detecting Human-to-Human Transmission of Avian Influenza A (H5N1). Emerging Infectious Disease journal 13:1348.

Yang, Y., J. D. Sugimoto, M. E. Halloran, N. E. Basta, D. L. Chao, L. Matrajt, G. Potter, E. Kenah, and I. M. Longini. 2009. The transmissibility and control of pandemic influenza A (H1N1) virus. Science (New York, N.Y.) 326:729–33.

Yang, Y., Y. Zhang, L. Fang, M. E. Halloran, M. Ma, S. Liang, E. Kenah, T. Britton, E. Chen, J. Hu, F. Tang, W. Cao, Z. Feng, and I. M. Longini. 2015. Household transmissibility of avian influenza A (H7N9) virus, China, February to May 2013 and October 2013 to March 2014. Euro surveillance : bulletin Européen sur les maladies transmissibles = European communicable disease bulletin 20.
