# Supplementary material for: Mapping influenza transmission in the ferret model to transmission in humans
Source: eLife. 2015 Sep 2;4:e07969. doi: 10.7554/eLife.07969 (PMC4586390; doi:10.7554/eLife.07969)
Supplement: Figure 1—source data 2. — DOI: http://dx.doi.org/10.7554/eLife.07969.005 [file elife07969s002.docx]

**Figure 1 – source data 2:** Ferret influenza transmission studies via respiratory droplets using human isolates. Transmission was determined using seroconversion (SC) and/or viral isolation (VI).

| **Subtype** | **Isolate** | **Successful transmissions – SC/VI (VI only)** | **Ferrets exposed** | **Reference** |
| --- | --- | --- | --- | --- |
| **H1N1** | A/Kawasaki/UTK-4/2009 | **1** | **2** | (Itoh et al. 2009) |
|  | A/Brisbane/59/2007 | **3** | **3** | (Maines et al. 2009) |
|  | A/Brisbane/59/2007 | **1** | **1** | (The SJCEIRS Working Group 2013) |
|  | A/Netherlands/26/2007 | **4 (3)** | **4** | (Munster et al. 2009) |
|  | A/South Dakota/6/2007 | **3** | **3** | (Pearce et al. 2011) |
|  | A/Solomon Islands/3/2006 | **3** | **3** | (Van Hoeven et al. 2009) |
|  | A/Texas/36/1991 | **3** | **3** | (Tumpey et al. 2007) |
| **H3N2** | A/Texas/50/2012 | **3** | **3** | (Belser et al. 2013) |
|  | A/Perth/16/2009 | **3 (2)** | **3** | (Houser et al. 2013) |
|  | A/Netherlands/213/2003 | **4** | **4** | (Munster et al. 2009) |
|  | A/Wyoming/3/2003**^RG^** | **2** | **2** | (Langlois et al. 2013) |
|  | A/Wyoming/3/2003 **^RG^** | **2** | **2** | (Jackson et al. 2009) |
|  | A/Panama/2007/1999 | **2** | **2** | (Gustin et al. 2011) |
|  | A/Panama/2007/1999 | **3** | **3** | (Maines et al. 2006) |
|  | A/Memphis/14/1998 | **2** | **2** | (Wan et al. 2008) |
|  | A/Wuhan/359/1995**^RG^** | **2** | **3** | (Yen et al. 2011) |
|  | A/Victoria/3/1975 | **3** | **3** | (Maines et al. 2006) |
|  | A/Victoria/3/1975 | **2** | **2** | (Itoh et al. 2009) |
| **pH1N1** | A/Shizuoka-c/99/2013 | **1** | **3** | (Takashita et al. 2015) |
|  | A/California/04/2009 | **3** | **3** | (Itoh et al. 2009) |
|  | A/California/04/2009 | **2** | **3** | (Maines et al. 2009) |
|  | A/California/04/2009**^RG^** | **1** | **1** | (Seibert et al. 2010) |
|  | A/California/04/2009**^RG^** | **2** | **3** | (Jayaraman et al. 2011) |
|  | A/California/04/2009 | **3** | **3** | (Yen et al. 2011) |
|  | A/California/04/2009**^RG^** | **3** | **3** | (Imai et al. 2012) |
|  | A/California/04/2009**^RG^** | **2** | **2** | (Martínez-Romero et al. 2013) |
|  | A/California/04/2009 | **3** | **3** | (Watanabe et al. 2013) |
|  | A/California/04/2009**^RG^** | **2** | **2** | (Hale et al. 2010) |
|  | A/California/04/2009 | **2** | **2** | (Koster et al. 2012) |
|  | A/California/04/2009**^RG^** | **2** | **2** | (Wong et al. 2012) |
|  | A/California/04/2009**^RG^** | **2** | **2** | (Karlsson et al. 2015) |
|  | A/California/07/2009 | **3** | **3** | (Zhu et al. 2013) |
|  | A/California/07/2009**^RG^** | **4** | **4** | (Lakdawala et al. 2011) |
|  | A/California/07/2009 | **3** | **3** | (Yuan et al. 2015) |
|  | A/Denmark/524/2009 | **2 (1)** | **2** | (Duan et al. 2010) |
|  | A/England/195/2009**^RG^** | **2** | **3** | (van Doremalen et al. 2011) |
|  | A/England/195/2009**^RG^** | **3** | **3** | (van Doremalen et al. 2011) |
|  | A/HH/01/2009**^RG^** | **1** | **1** | (Seibert et al. 2010) |
|  | A/HK/415742/2009 | **2** | **3** | (Yen et al. 2011) |
|  | A/Mexico/4482/2009 | **2** | **3** | (Maines et al. 2009) |
|  | A/Netherlands/602/2009 | **4** | **4** | (Munster et al. 2009) |
|  | A/Netherlands/602/2009**^RG^** | **4** | **4** | (Herfst et al. 2010) |
|  | A/Netherlands/602/2009**^RG^** | **4** | **4** | (Chutinimitkul et al. 2010) |
|  | A/Netherlands/602/2009**^RG^** | **4** | **4** | (van der Vries et al. 2011) |
|  | A/Netherlands/1132/2009 | **3** | **3** | (Pearce et al. 2011) |
|  | A/New York/1682/2009**^RG^** | **2** | **3** | (Zhou et al. 2013) |
|  | A/Osaka/164/2009 | **3** | **3** | (Kiso et al. 2010) |
|  | A/Quebec/144147/2009**^RG^** | **3 (1)** | **4** | (Pizzorno et al. 2012) |
|  | A/Quebec/147023/2009 | **4** | **4** | (Hamelin et al. 2011) |
|  | A/Sichuan/1/2009 | **3** | **3** | (Zhang et al. 2012) |
|  | A/Texas/15/2009 | **2** | **3** | (Maines et al. 2009) |
|  | A/Vietnam/HCM9727/2009 | **3** | **3** | (Kiso et al. 2010) |
| **H2N2** | A/England/10/1967 | **3** | **3** | (Pappas et al. 2015) |
|  | A/Albany/6/1958 | **3** | **3** | (Pappas et al. 2010) |
|  | A/El Salvador/2/1957 | **1** | **3** | (Pappas et al. 2010) |
| **H7N9** | A/Anhui/1/2013 | **2** | **6** | (Belser et al. 2013) |
|  | A/Anhui/1/2013 | **3** | **4** | (Richard et al. 2013) |
|  | A/Anhui/1/2013 | **1** | **3** | (Watanabe et al. 2013) |
|  | A/Anhui/1/2013 | **3** | **3** | (Zhang et al. 2013) |
|  | A/Anhui/1/2013 | **3** | **3** | (Zhang et al. 2013) |
|  | A/Anhui/1/2013 | **1** | **3** | (Xu et al. 2014) |
|  | A/Anhui/1/2013 | **2 (1)** | **4** | (Zaraket et al. 2015) |
|  | A/Shanghai/1/2013 | **1** | **3** | (Belser et al. 2013) |
|  | A/Shanghai/1/2013 | **1** | **3** | (Zhang et al. 2013) |
|  | A/Shanghai/1/2013 | **4** | **4** | (Yen et al. 2014) |
|  | A/Shanghai/2/2013 | **1** | **3** | (Zhang et al. 2013) |
|  | A/Shanghai/2/2013 | **2 (1)** | **3** | (Zhu et al. 2013) |
| **H5N1** | A/Indonesia/5/2005 | **0** | **4** | (Munster et al. 2009) |
|  | A/Thailand/16/2004**^RG^** | **0** | **3** | (Jackson et al. 2009) |
|  | A/Vietnam/1194/2004 | **0** | **3** | (Xu et al. 2013) |
|  | A/Vietnam/1203/2004**^RG^** | **0** | **3** | (Maines et al. 2011) |
|  | A/Vietnam/1203/2004 | **0** | **3** | (Xu et al. 2014) |
|  | A/Hong Kong/213/2003 | **0** | **3** | (Maines et al. 2006) |
|  | A/Hong Kong/486/1997 | **2 (0)** | **3** | (Maines et al. 2006) |
|  | A/Hong Kong/486/1997**^RG^** | **2 (0)** | **3** | (Maines et al. 2011) |
| **H7N2** | A/NY/107/2003 | **0** | **3** | (Belser et al. 2008) |
| **H7N7** | A/NL/219/2003 | **0** | **3** | (Belser et al. 2008) |
|  | A/NL/230/2003 | **0** | **3** | (Belser et al. 2008) |
| **H9N2** | A/Hong Kong/33982/2009 | **0** | **2** | (The SJCEIRS Working Group 2013) |
|  | A/Hong Kong/1073/1999 | **0** | **2** | (The SJCEIRS Working Group 2013) |
| **H7N3** | A/Canada/504/2004 | **0** | **3** | (Belser et al. 2008) |

**^RG^**Isolate rescued using reverse genetics.

**References**

Belser, J. A., O. Blixt, L.-M. Chen, C. Pappas, T. R. Maines, N. Van Hoeven, R. Donis, J. Busch, R. McBride, J. C. Paulson, J. M. Katz, and T. M. Tumpey. 2008. Contemporary North American influenza H7 viruses possess human receptor specificity: Implications for virus transmissibility. Proceedings of the National Academy of Sciences of the United States of America 105:7558–63.

Belser, J. A., K. M. Gustin, M. B. Pearce, T. R. Maines, H. Zeng, C. Pappas, X. Sun, P. J. Carney, J. M. Villanueva, J. Stevens, J. M. Katz, and T. M. Tumpey. 2013. Pathogenesis and transmission of avian influenza A (H7N9) virus in ferrets and mice. Nature 501:556–9.

Chutinimitkul, S., S. Herfst, J. Steel, A. C. Lowen, J. Ye, D. van Riel, E. J. A. Schrauwen, T. M. Bestebroer, B. Koel, D. F. Burke, K. H. Sutherland-Cash, C. S. Whittleston, C. A. Russell, D. J. Wales, D. J. Smith, M. Jonges, A. Meijer, M. Koopmans, G. F. Rimmelzwaan, T. Kuiken, A. D. M. E. Osterhaus, A. Garcia-Sastre, D. R. Perez, and R. A. M. Fouchier. 2010. Virulence-Associated Substitution D222G in the Hemagglutinin of 2009 Pandemic Influenza A(H1N1) Virus Affects Receptor Binding. Journal of Virology 84:11802–11813.

Van Doremalen, N., H. Shelton, K. L. Roberts, I. M. Jones, R. J. Pickles, C. I. Thompson, and W. S. Barclay. 2011. A single amino acid in the HA of pH1N1 2009 influenza virus affects cell tropism in human airway epithelium, but not transmission in ferrets. PloS one 6:e25755.

Duan, S., D. A. Boltz, P. Seiler, J. Li, K. Bragstad, L. P. Nielsen, R. J. Webby, R. G. Webster, and E. A. Govorkova. 2010. Oseltamivir-resistant pandemic H1N1/2009 influenza virus possesses lower transmissibility and fitness in ferrets. PLoS pathogens 6:e1001022.

Gustin, K. M., J. A. Belser, D. A. Wadford, M. B. Pearce, J. M. Katz, T. M. Tumpey, and T. R. Maines. 2011. Influenza virus aerosol exposure and analytical system for ferrets. Proceedings of the National Academy of Sciences of the United States of America 108:8432–7.

Hale, B. G., J. Steel, B. Manicassamy, R. A. Medina, J. Ye, D. Hickman, A. C. Lowen, D. R. Perez, and A. García-Sastre. 2010. Mutations in the NS1 C-terminal tail do not enhance replication or virulence of the 2009 pandemic H1N1 influenza A virus. The Journal of general virology 91:1737–42.

Hamelin, M.-E., M. Baz, X. Bouhy, E. Beaulieu, K. Dubé, C. Mallett, and G. Boivin. 2011. Reduced airborne transmission of oseltamivir-resistant pandemic A/H1N1 virus in ferrets. Antiviral therapy 16:775–9.

Herfst, S., S. Chutinimitkul, J. Ye, E. de Wit, V. J. Munster, E. J. A. Schrauwen, T. M. Bestebroer, M. Jonges, A. Meijer, M. Koopmans, G. F. Rimmelzwaan, A. D. M. E. Osterhaus, D. R. Perez, and R. A. M. Fouchier. 2010. Introduction of virulence markers in PB2 of pandemic swine-origin influenza virus does not result in enhanced virulence or transmission. Journal of virology 84:3752–8.

Van Hoeven, N., C. Pappas, J. A. Belser, T. R. Maines, H. Zeng, A. García-Sastre, R. Sasisekharan, J. M. Katz, and T. M. Tumpey. 2009. Human HA and polymerase subunit PB2 proteins confer transmission of an avian influenza virus through the air. Proceedings of the National Academy of Sciences of the United States of America 106:3366–71.

Houser, K. V, M. B. Pearce, J. M. Katz, and T. M. Tumpey. 2013. Impact of prior seasonal H3N2 influenza vaccination or infection on protection and transmission of emerging variants of influenza A(H3N2)v virus in ferrets. Journal of virology 87:13480–9.

Imai, M., T. Watanabe, M. Hatta, S. C. Das, M. Ozawa, K. Shinya, G. Zhong, A. Hanson, H. Katsura, S. Watanabe, C. Li, E. Kawakami, S. Yamada, M. Kiso, Y. Suzuki, E. A. Maher, G. Neumann, and Y. Kawaoka. 2012. Experimental adaptation of an influenza H5 HA confers respiratory droplet transmission to a reassortant H5 HA/H1N1 virus in ferrets. Nature 486:420–8.

Itoh, Y., K. Shinya, M. Kiso, T. Watanabe, Y. Sakoda, M. Hatta, Y. Muramoto, D. Tamura, Y. Sakai-Tagawa, T. Noda, S. Sakabe, M. Imai, Y. Hatta, S. Watanabe, C. Li, S. Yamada, K. Fujii, S. Murakami, H. Imai, S. Kakugawa, M. Ito, R. Takano, K. Iwatsuki-Horimoto, M. Shimojima, T. Horimoto, H. Goto, K. Takahashi, A. Makino, H. Ishigaki, M. Nakayama, M. Okamatsu, K. Takahashi, D. Warshauer, P. A. Shult, R. Saito, H. Suzuki, Y. Furuta, M. Yamashita, K. Mitamura, K. Nakano, M. Nakamura, R. Brockman-Schneider, H. Mitamura, M. Yamazaki, N. Sugaya, M. Suresh, M. Ozawa, G. Neumann, J. Gern, H. Kida, K. Ogasawara, and Y. Kawaoka. 2009. In vitro and in vivo characterization of new swine-origin H1N1 influenza viruses. Nature 460:1021–5.

Jackson, S., N. Van Hoeven, L.-M. Chen, T. R. Maines, N. J. Cox, J. M. Katz, and R. O. Donis. 2009. Reassortment between avian H5N1 and human H3N2 influenza viruses in ferrets: a public health risk assessment. Journal of virology 83:8131–40.

Jayaraman, A., C. Pappas, R. Raman, J. A. Belser, K. Viswanathan, Z. Shriver, T. M. Tumpey, and R. Sasisekharan. 2011. A single base-pair change in 2009 H1N1 hemagglutinin increases human receptor affinity and leads to efficient airborne viral transmission in ferrets. PloS one 6:e17616.

Karlsson, E. A., V. A. Meliopoulos, C. Savage, B. Livingston, A. Mehle, and S. Schultz-Cherry. 2015. Visualizing real-time influenza virus infection, transmission and protection in ferrets. Nature communications 6:6378.

Kiso, M., K. Shinya, M. Shimojima, R. Takano, K. Takahashi, H. Katsura, S. Kakugawa, M. T. Q. Le, M. Yamashita, Y. Furuta, M. Ozawa, and Y. Kawaoka. 2010. Characterization of oseltamivir-resistant 2009 H1N1 pandemic influenza A viruses. PLoS pathogens 6:e1001079.

Koster, F., K. Gouveia, Y. Zhou, K. Lowery, R. Russell, H. MacInnes, Z. Pollock, R. C. Layton, J. Cromwell, D. Toleno, J. Pyle, M. Zubelewicz, K. Harrod, R. Sampath, S. Hofstadler, P. Gao, Y. Liu, and Y.-S. Cheng. 2012. Exhaled aerosol transmission of pandemic and seasonal H1N1 influenza viruses in the ferret. PloS one 7:e33118.

Lakdawala, S. S., E. W. Lamirande, A. L. Suguitan, W. Wang, C. P. Santos, L. Vogel, Y. Matsuoka, W. G. Lindsley, H. Jin, and K. Subbarao. 2011. Eurasian-origin gene segments contribute to the transmissibility, aerosol release, and morphology of the 2009 pandemic H1N1 influenza virus. PLoS pathogens 7:e1002443.

Langlois, R. A., R. A. Albrecht, B. Kimble, T. Sutton, J. S. Shapiro, C. Finch, M. Angel, M. A. Chua, A. S. Gonzalez-Reiche, K. Xu, D. Perez, A. García-Sastre, and B. R. tenOever. 2013. MicroRNA-based strategy to mitigate the risk of gain-of-function influenza studies. Nature biotechnology 31:844–7.

Maines, T. R., L.-M. Chen, N. Van Hoeven, T. M. Tumpey, O. Blixt, J. A. Belser, K. M. Gustin, M. B. Pearce, C. Pappas, J. Stevens, N. J. Cox, J. C. Paulson, R. Raman, R. Sasisekharan, J. M. Katz, and R. O. Donis. 2011. Effect of receptor binding domain mutations on receptor binding and transmissibility of avian influenza H5N1 viruses. Virology 413:139–47.

Maines, T. R., L.-M. Chen, Y. Matsuoka, H. Chen, T. Rowe, J. Ortin, A. Falcón, T. H. Nguyen, L. Q. Mai, E. R. Sedyaningsih, S. Harun, T. M. Tumpey, R. O. Donis, N. J. Cox, K. Subbarao, and J. M. Katz. 2006. Lack of transmission of H5N1 avian-human reassortant influenza viruses in a ferret model. Proceedings of the National Academy of Sciences of the United States of America 103:12121–6.

Maines, T. R., A. Jayaraman, J. A. Belser, D. A. Wadford, C. Pappas, H. Zeng, K. M. Gustin, M. B. Pearce, K. Viswanathan, Z. H. Shriver, R. Raman, N. J. Cox, R. Sasisekharan, J. M. Katz, and T. M. Tumpey. 2009. Transmission and pathogenesis of swine-origin 2009 A(H1N1) influenza viruses in ferrets and mice. Science (New York, N.Y.) 325:484–7.

Martínez-Romero, C., E. de Vries, A. Belicha-Villanueva, I. Mena, D. M. Tscherne, V. L. Gillespie, R. A. Albrecht, C. A. M. de Haan, and A. García-Sastre. 2013. Substitutions T200A and E227A in the hemagglutinin of pandemic 2009 influenza A virus increase lethality but decrease transmission. Journal of virology 87:6507–11.

Munster, V. J., E. de Wit, J. M. A. van den Brand, S. Herfst, E. J. A. Schrauwen, T. M. Bestebroer, D. van de Vijver, C. A. Boucher, M. Koopmans, G. F. Rimmelzwaan, T. Kuiken, A. D. M. E. Osterhaus, and R. A. M. Fouchier. 2009. Pathogenesis and transmission of swine-origin 2009 A(H1N1) influenza virus in ferrets. Science (New York, N.Y.) 325:481–3.

Pappas, C., K. Viswanathan, A. Chandrasekaran, R. Raman, J. M. Katz, R. Sasisekharan, and T. M. Tumpey. 2010. Receptor Specificity and Transmission of H2N2 Subtype Viruses Isolated from the Pandemic of 1957. PLoS ONE 5:e11158.

Pappas, C., H. Yang, P. J. Carney, M. B. Pearce, J. M. Katz, J. Stevens, and T. M. Tumpey. 2015. Assessment of transmission, pathogenesis and adaptation of H2 subtype influenza viruses in ferrets. Virology 477:61–71.

Pearce, M. B., J. A. Belser, K. V Houser, J. M. Katz, and T. M. Tumpey. 2011. Efficacy of seasonal live attenuated influenza vaccine against virus replication and transmission of a pandemic 2009 H1N1 virus in ferrets. Vaccine 29:2887–94.

Pizzorno, A., Y. Abed, X. Bouhy, E. Beaulieu, C. Mallett, R. Russell, and G. Boivin. 2012. Impact of mutations at residue I223 of the neuraminidase protein on the resistance profile, replication level, and virulence of the 2009 pandemic influenza virus. Antimicrobial agents and chemotherapy 56:1208–14.

Richard, M., E. J. A. Schrauwen, M. de Graaf, T. M. Bestebroer, M. I. J. Spronken, S. van Boheemen, D. de Meulder, P. Lexmond, M. Linster, S. Herfst, D. J. Smith, J. M. van den Brand, D. F. Burke, T. Kuiken, G. F. Rimmelzwaan, A. D. M. E. Osterhaus, and R. A. M. Fouchier. 2013. Limited airborne transmission of H7N9 influenza A virus between ferrets. Nature 501:560–3.

Seibert, C. W., M. Kaminski, J. Philipp, D. Rubbenstroth, R. a Albrecht, F. Schwalm, S. Stertz, R. a Medina, G. Kochs, A. García-Sastre, P. Staeheli, and P. Palese. 2010. Oseltamivir-resistant variants of the 2009 pandemic H1N1 influenza A virus are not attenuated in the guinea pig and ferret transmission models. Journal of virology 84:11219–26.

Takashita, E., M. Kiso, S. Fujisaki, M. Yokoyama, K. Nakamura, M. Shirakura, H. Sato, T. Odagiri, Y. Kawaoka, and M. Tashiro. 2015. Characterization of a large cluster of influenza A(H1N1)pdm09 viruses cross-resistant to oseltamivir and peramivir during the 2013-2014 influenza season in Japan. Antimicrobial agents and chemotherapy 59:2607–17.

The SJCEIRS Working Group. 2013. Assessing the fitness of distinct clades of influenza A (H9N2) viruses. Emerging Microbes & Infections 2:e75.

Tumpey, T. M., T. R. Maines, N. Van Hoeven, L. Glaser, A. Solórzano, C. Pappas, N. J. Cox, D. E. Swayne, P. Palese, J. M. Katz, and A. García-Sastre. 2007. A two-amino acid change in the hemagglutinin of the 1918 influenza virus abolishes transmission. Science 315:655–9.

Van der Vries, E., E. J. Veldhuis Kroeze, K. J. Stittelaar, M. Linster, A. Van der Linden, E. J. A. Schrauwen, L. M. Leijten, G. van Amerongen, M. Schutten, T. Kuiken, A. D. M. E. Osterhaus, R. A. M. Fouchier, C. A. B. Boucher, and S. Herfst. 2011. Multidrug resistant 2009 A/H1N1 influenza clinical isolate with a neuraminidase I223R mutation retains its virulence and transmissibility in ferrets. PLoS pathogens 7:e1002276.

Wan, H., E. M. Sorrell, H. Song, M. J. Hossain, G. Ramirez-Nieto, I. Monne, J. Stevens, G. Cattoli, I. Capua, L.-M. Chen, R. O. Donis, J. Busch, J. C. Paulson, C. Brockwell, R. Webby, J. Blanco, M. Q. Al-Natour, and D. R. Perez. 2008. Replication and transmission of H9N2 influenza viruses in ferrets: evaluation of pandemic potential. PloS one 3:e2923.

Watanabe, T., M. Kiso, S. Fukuyama, N. Nakajima, M. Imai, S. Yamada, S. Murakami, S. Yamayoshi, K. Iwatsuki-Horimoto, Y. Sakoda, E. Takashita, R. McBride, T. Noda, M. Hatta, H. Imai, D. Zhao, N. Kishida, M. Shirakura, R. P. de Vries, S. Shichinohe, M. Okamatsu, T. Tamura, Y. Tomita, N. Fujimoto, K. Goto, H. Katsura, E. Kawakami, I. Ishikawa, S. Watanabe, M. Ito, Y. Sakai-Tagawa, Y. Sugita, R. Uraki, R. Yamaji, A. J. Eisfeld, G. Zhong, S. Fan, J. Ping, E. A. Maher, A. Hanson, Y. Uchida, T. Saito, M. Ozawa, G. Neumann, H. Kida, T. Odagiri, J. C. Paulson, H. Hasegawa, M. Tashiro, and Y. Kawaoka. 2013. Characterization of H7N9 influenza A viruses isolated from humans. Nature 501:551–5.

Wong, D. D. Y., K.-T. Choy, R. W. Y. Chan, S. F. Sia, H.-P. Chiu, P. P. H. Cheung, M. C. W. Chan, J. S. M. Peiris, and H.-L. Yen. 2012. Comparable fitness and transmissibility between oseltamivir-resistant pandemic 2009 and seasonal H1N1 influenza viruses with the H275Y neuraminidase mutation. Journal of virology 86:10558–70.

Xu, L., L. Bao, W. Deng, L. Dong, H. Zhu, T. Chen, Q. Lv, F. Li, J. Yuan, Z. Xiang, K. Gao, Y. Xu, L. Huang, Y. Li, J. Liu, Y. Yao, P. Yu, X. Li, W. Huang, X. Zhao, Y. Lan, J. Guo, W. Yong, Q. Wei, H. Chen, L. Zhang, and C. Qin. 2014. Novel avian-origin human influenza A(H7N9) can be transmitted between ferrets via respiratory droplets. The Journal of infectious diseases 209:551–6.

Xu, L., L. Bao, J. Yuan, F. Li, Q. Lv, W. Deng, Y. Xu, Y. Yao, P. Yu, H. Chen, K.-Y. Yuen, and C. Qin. 2013. Antigenicity and transmissibility of a novel clade 2.3.2.1 avian influenza H5N1 virus. The Journal of general virology 94:2616–26.

Yen, H.-L., C.-H. Liang, C.-Y. Wu, H. L. Forrest, A. Ferguson, K.-T. Choy, J. Jones, D. D.-Y. Wong, P. P.-H. Cheung, C.-H. Hsu, O. T. Li, K. M. Yuen, R. W. Y. Chan, L. L. M. Poon, M. C. W. Chan, J. M. Nicholls, S. Krauss, C.-H. Wong, Y. Guan, R. G. Webster, R. J. Webby, and M. Peiris. 2011. Hemagglutinin-neuraminidase balance confers respiratory-droplet transmissibility of the pandemic H1N1 influenza virus in ferrets. Proceedings of the National Academy of Sciences of the United States of America 108:14264–9.

Yen, H.-L., J. Zhou, K.-T. Choy, S. F. Sia, O. Teng, I. H. Ng, V. J. Fang, Y. Hu, W. Wang, B. J. Cowling, J. M. Nicholls, Y. Guan, and J. S. M. Peiris. 2014. The R292K Mutation That Confers Resistance to Neuraminidase Inhibitors Leads to Competitive Fitness Loss of A/Shanghai/1/2013 (H7N9) Influenza Virus in Ferrets. Journal of Infectious Diseases 210:1900–1908.

Yuan, J., L. Xu, L. Bao, Y. Yao, W. Deng, F. Li, Q. Lv, S. Gu, Q. Wei, and C. Qin. 2015. Characterization of an H9N2 avian influenza virus from a Fringilla montifringilla brambling in northern China. Virology 476:289–297.

Zaraket, H., T. Baranovich, B. S. Kaplan, R. Carter, M.-S. Song, J. C. Paulson, J. E. Rehg, J. Bahl, J. C. Crumpton, J. Seiler, M. Edmonson, G. Wu, E. Karlsson, T. Fabrizio, H. Zhu, Y. Guan, M. Husain, S. Schultz-Cherry, S. Krauss, R. McBride, R. G. Webster, E. A. Govorkova, J. Zhang, C. J. Russell, and R. J. Webby. 2015. Mammalian adaptation of influenza A(H7N9) virus is limited by a narrow genetic bottleneck. Nature communications 6:6553.

Zhang, Q., J. Shi, G. Deng, J. Guo, X. Zeng, X. He, H. Kong, C. Gu, X. Li, J. Liu, G. Wang, Y. Chen, L. Liu, L. Liang, Y. Li, J. Fan, J. Wang, W. Li, L. Guan, Q. Li, H. Yang, P. Chen, L. Jiang, Y. Guan, X. Xin, Y. Jiang, G. Tian, X. Wang, C. Qiao, C. Li, Z. Bu, and H. Chen. 2013. H7N9 influenza viruses are transmissible in ferrets by respiratory droplet. Science (New York, N.Y.) 341:410–4.

Zhang, Y., Q. Zhang, Y. Gao, X. He, H. Kong, Y. Jiang, Y. Guan, X. Xia, Y. Shu, Y. Kawaoka, Z. Bu, and H. Chen. 2012. Key molecular factors in hemagglutinin and PB2 contribute to efficient transmission of the 2009 H1N1 pandemic influenza virus. Journal of virology 86:9666–74.

Zhou, B., M. B. Pearce, Y. Li, J. Wang, R. J. Mason, T. M. Tumpey, and D. E. Wentworth. 2013. Asparagine substitution at PB2 residue 701 enhances the replication, pathogenicity, and transmission of the 2009 pandemic H1N1 influenza A virus. PloS one 8:e67616.

Zhu, H., D. Wang, D. J. Kelvin, L. Li, Z. Zheng, S.-W. Yoon, S.-S. Wong, A. Farooqui, J. Wang, D. Banner, R. Chen, R. Zheng, J. Zhou, Y. Zhang, W. Hong, W. Dong, Q. Cai, M. H. A. Roehrl, S. S. H. Huang, A. A. Kelvin, T. Yao, B. Zhou, X. Chen, G. M. Leung, L. L. M. Poon, R. G. Webster, R. J. Webby, J. S. M. Peiris, Y. Guan, and Y. Shu. 2013. Infectivity, transmission, and pathology of human-isolated H7N9 influenza virus in ferrets and pigs. Science (New York, N.Y.) 341:183–6.
