# Supplementary material for: Mapping influenza transmission in the ferret model to transmission in humans
Source: eLife. 2015 Sep 2;4:e07969. doi: 10.7554/eLife.07969 (PMC4586390; doi:10.7554/eLife.07969)
Supplement: Figure 1—source data 3. — DOI: http://dx.doi.org/10.7554/eLife.07969.006 [file elife07969s003.docx]

**Figure 1 – source data 3:** Ferret influenza transmission studies via direct contact using human isolates. Transmission was determined using seroconversion (SC) and/or viral isolation (VI).

.

| **Subtype** | **Isolate** | | **Successful transmissions – SC/VI (VI only)** | **Ferrets exposed** | **Reference** |
| --- | --- | --- | --- | --- | --- |
| **H1N1** | A/Brisbane/59/2007 | | **3** | **3** | (Maines et al. 2009) |
|  | A/Brisbane/59/2007 | | **2** | **2** | (Hurt et al. 2010) |
|  | A/Brisbane/59/2007 | | **1** | **1** | (The SJCEIRS Working Group 2013) |
|  | A/Brisbane/59/2007 | | **4** | **4** | (Abed et al. 2011) |
|  | A/Brisbane/59/2007 | | **4** | **4** | (Ellebedy et al. 2011) |
|  | A/New Caledonia-like | | **12** | **12** | (Herlocher et al. 2004) |
| **H3N2** | A/Texas/50/2012 | | **3** | **3** | (Belser et al. 2013) |
|  | A/Brisbane/10/2007 | | **2** | **2** | (Lee et al. 2013) |
|  | A/Bethesda/NIH12- 0/2008 | | **3** | **4** | (Memoli et al. 2010) |
|  | A/Fukui/20/2004 | | **2** | **2** | (Hurt et al. 2010) |
|  | A/Wyoming/3/2003**^RG^** | | **2** | **2** | (Langlois et al. 2013) |
|  | A/Panama/2007/1999 | | **2** | **2** | (Gustin et al. 2011) |
|  | A/Memphis/14/1998 | | **2** | **2** | (Wan et al. 2008) |
|  | A/Sydney/5/1997 | | **5** | **5** | (Herlocher et al. 2001) |
|  | A/Sydney/5/1997 | | **12** | **12** | (Herlocher et al. 2002) |
|  | A/Sydney/5/1997 | | **3** | **4** | (Oxford et al. 2007) |
|  | A/Sydney/5/1997 | | **4 (1)** | **4** | (Oxford et al. 2007) |
|  | A/Wuhan/359/1995 | | **11** | **11** | (Herlocher et al. 2004) |
|  | A/Wuhan/359/1995**^RG^** | | **2** | **2** | (Yen et al. 2005) |
|  | A/Wuhan/359/1995**^RG^** | | **3** | **3** | (Yen et al. 2011) |
|  | A/LA/1/1987 | | **5** | **5** | (Herlocher et al. 2001) |
|  | A/Victoria/3/1975**^RG^** | | **6 (5)** | **6** | (Roberts et al. 2011) |
| **pH1N1** | A/Bethesda/NIH106-D0/2009 | | **4** | **4** | (Memoli et al. 2011) |
|  | A/Bethesda/NIH107-D0/2009 | | **4** | **4** | (Memoli et al. 2011) |
|  | A/California/04/2009 | | **3** | **3** | (Maines et al. 2009) |
|  | A/California/04/2009**^RG^** | | **1** | **1** | (Seibert et al. 2010) |
|  | A/California/04/2009 | | **3** | **3** | (Yen et al. 2011) |
|  | A/California/04/2009**^RG^** | | **3** | **3** | (Zanin et al. 2015) |
|  | A/California/04/2009**^RG^** | | **3** | **3** | (Karlsson et al. 2015) |
|  | A/California/04/2009 | | **2** | **2** | (Hale et al. 2010) |
|  | A/California/04/2009**^RG^** | | **2** | **2** | (Wong et al. 2012) |
|  | A/California/07/2009 | | **3** | **3** | (Zhu et al. 2013) |
|  | A/California/07/2009 | | **3** | **3** | (Oh et al. 2014) |
|  | A/California/07/2009 | | **3** | **3** | (Oh et al. 2014) |
|  | A/California/07/2009 | | **12** | **12** | (Oh et al. 2014) |
|  | A/California/07/2009 | | **12** | **12** | (Oh et al. 2014) |
|  | A/Denmark/524/2009 | | **2** | **2** | (Duan et al. 2010) |
|  | A/England/195/2009**^RG^** | | **3** | **3** | (van Doremalen et al. 2011) |
|  | A/England/195/2009**^RG^** | | **2** | **2** | (Roberts et al. 2012) |
|  | A/England/195/2009**^RG^** | | **3** | **3** | (Roberts et al. 2012) |
|  | A/HH/01/2009**^RG^** | | **1** | **1** | (Seibert et al. 2010) |
|  | A/HK/415742/2009 | | **3** | **3** | (Yen et al. 2011) |
|  | A/Mexico/4482/2009 | | **3** | **3** | (Maines et al. 2009) |
|  | A/Quebec/144147/2009**^RG^** | | **4** | **4** | (Pizzorno et al. 2012) |
|  | A/Quebec/144147/2009**^RG^** | | **4** | **4** | (Abed et al. 2014) |
|  | A/Quebec/147023/2009 | | **4** | **4** | (Hamelin et al. 2011) |
|  | A/Tasmania/2004/2009 | | **7** | **8** | (Guarnaccia et al. 2013) |
|  | A/Tennessee/1-560/2009 | | **4 (3)** | **4** | (Ellebedy et al. 2011) |
|  | A/Texas/15/2009 | | **3** | **3** | (Maines et al. 2009) |
| **H2N2** | A/England/10/1967 | | **3** | **3** | (Pappas et al. 2015) |
|  | | A/Albany/6/1958 | **3** | **3** | (Pappas et al. 2010) |
|  | | A/El Salvador/2/1957 | **1 (0)** | **3** | (Pappas et al. 2010) |
| **H7N9** | A/Anhui/1/2013 | | **4** | **4** | (Belser et al. 2013) |
|  | A/Anhui/1/2013 | | **4 (3)** | **4** | (Zaraket et al. 2015) |
|  | A/Shanghai/1/2013 | | **4** | **4** | (Belser et al. 2013) |
|  | A/Shanghai/1/2013 | | **4** | **4** | (Yen et al. 2014) |
|  | A/Shanghai/2/2013 | | **3** | **3** | (Zhu et al. 2013) |
| **H5N1** | A/Turkey/65-596/2006 | | **0** | **2** | (Yen et al. 2007) |
|  | A/Indonesia/5/2005 | | **0** | **3** | (Maines et al. 2006) |
|  | A/Vietnam/HN30408/2005 | | **0** | **3** | (Maines et al. 2006) |
|  | A/Vietnam/JP36-2/2005 | | **2** | **4** | (Yen et al. 2007) |
|  | A/Thailand/16/2004**^RG^** | | **0** | **3** | (Jackson et al. 2009) |
|  | A/Vietnam/1203/2004 | | **0** | **4** | (Yen et al. 2007) |
|  | A/Vietnam/1203/2004**^RG^** | | **1 (0)** | **3** | (Maines et al. 2011) |
|  | A/Vietnam/1203/2004**^RG^** | | **0** | **4** | (Zaraket et al. 2013) |
|  | A/Hong Kong/213/2003 | | **1 (0)** | **4** | (Yen et al. 2007) |
|  | A/Hong Kong/486/1997 | | **2 (1)** | **2** | (Maines et al. 2006) |
|  | A/Hong Kong/486/1997**^RG^** | | **2 (1)** | **2** | (Maines et al. 2011) |
| **H7N2** | A/NY/107/2003 | | **3** | **3** | (Belser et al. 2008) |
| **H7N7** | A/NL/219/2003 | | **0** | **3** | (Belser et al. 2008) |
|  | A/NL/230/2003 | | **2** | **3** | (Belser et al. 2008) |
|  | A/NL/230/2003 | | **2** | **3** | (Belser et al. 2008) |
| **H9N2** | A/Hong Kong/33982/2009 | | **1** | **2** | (The SJCEIRS Working Group 2013) |
|  | A/Hong Kong/1073/1999 | | **1** | **2** | (The SJCEIRS Working Group 2013) |
| **H7N3** | A/Mexico/InDRE7218/2012 | | **3** | **3** | (Belser et al. 2013) |
|  | A/Canada/504/2004 | | **2** | **3** | (Belser et al. 2008) |

**^RG^**Isolate rescued using reverse genetics.

**References**

Abed, Y., A. Pizzorno, X. Bouhy, and G. Boivin. 2011. Role of permissive neuraminidase mutations in influenza A/Brisbane/59/2007-like (H1N1) viruses. PLoS pathogens 7:e1002431.

Abed, Y., A. Pizzorno, X. Bouhy, C. Rhéaume, and G. Boivin. 2014. Impact of potential permissive neuraminidase mutations on viral fitness of the H275Y oseltamivir-resistant influenza A(H1N1)pdm09 virus in vitro, in mice and in ferrets. Journal of virology 88:1652–8.

Belser, J. A., O. Blixt, L.-M. Chen, C. Pappas, T. R. Maines, N. Van Hoeven, R. Donis, J. Busch, R. McBride, J. C. Paulson, J. M. Katz, and T. M. Tumpey. 2008. Contemporary North American influenza H7 viruses possess human receptor specificity: Implications for virus transmissibility. Proceedings of the National Academy of Sciences of the United States of America 105:7558–63.

Belser, J. A., K. M. Gustin, M. B. Pearce, T. R. Maines, H. Zeng, C. Pappas, X. Sun, P. J. Carney, J. M. Villanueva, J. Stevens, J. M. Katz, and T. M. Tumpey. 2013. Pathogenesis and transmission of avian influenza A (H7N9) virus in ferrets and mice. Nature 501:556–9.

Van Doremalen, N., H. Shelton, K. L. Roberts, I. M. Jones, R. J. Pickles, C. I. Thompson, and W. S. Barclay. 2011. A single amino acid in the HA of pH1N1 2009 influenza virus affects cell tropism in human airway epithelium, but not transmission in ferrets. PloS one 6:e25755.

Duan, S., D. A. Boltz, P. Seiler, J. Li, K. Bragstad, L. P. Nielsen, R. J. Webby, R. G. Webster, and E. A. Govorkova. 2010. Oseltamivir-resistant pandemic H1N1/2009 influenza virus possesses lower transmissibility and fitness in ferrets. PLoS pathogens 6:e1001022.

Ellebedy, A. H., M. F. Ducatez, S. Duan, E. Stigger-Rosser, A. M. Rubrum, E. A. Govorkova, R. G. Webster, and R. J. Webby. 2011. Impact of prior seasonal influenza vaccination and infection on pandemic A (H1N1) influenza virus replication in ferrets. Vaccine 29:3335–9.

Guarnaccia, T., L. A. Carolan, S. Maurer-Stroh, R. T. C. Lee, E. Job, P. C. Reading, S. Petrie, J. M. McCaw, J. McVernon, A. C. Hurt, A. Kelso, J. Mosse, I. G. Barr, and K. L. Laurie. 2013. Antigenic drift of the pandemic 2009 A(H1N1) influenza virus in A ferret model. PLoS pathogens 9:e1003354.

Gustin, K. M., J. A. Belser, D. A. Wadford, M. B. Pearce, J. M. Katz, T. M. Tumpey, and T. R. Maines. 2011. Influenza virus aerosol exposure and analytical system for ferrets. Proceedings of the National Academy of Sciences of the United States of America 108:8432–7.

Hale, B. G., J. Steel, B. Manicassamy, R. A. Medina, J. Ye, D. Hickman, A. C. Lowen, D. R. Perez, and A. García-Sastre. 2010. Mutations in the NS1 C-terminal tail do not enhance replication or virulence of the 2009 pandemic H1N1 influenza A virus. The Journal of general virology 91:1737–42.

Hamelin, M.-E., M. Baz, X. Bouhy, E. Beaulieu, K. Dubé, C. Mallett, and G. Boivin. 2011. Reduced airborne transmission of oseltamivir-resistant pandemic A/H1N1 virus in ferrets. Antiviral therapy 16:775–9.

Herlocher, M. L., J. Carr, J. Ives, S. Elias, R. Truscon, N. Roberts, and A. S. Monto. 2002. Influenza virus carrying an R292K mutation in the neuraminidase gene is not transmitted in ferrets. Antiviral research 54:99–111.

Herlocher, M. L., S. Elias, R. Truscon, S. Harrison, D. Mindell, C. Simon, and A. S. Monto. 2001. Ferrets as a transmission model for influenza: sequence changes in HA1 of type A (H3N2) virus. The Journal of infectious diseases 184:542–6.

Herlocher, M. L., R. Truscon, S. Elias, H.-L. Yen, N. a Roberts, S. E. Ohmit, and A. S. Monto. 2004. Influenza viruses resistant to the antiviral drug oseltamivir: transmission studies in ferrets. The Journal of infectious diseases 190:1627–30.

Hurt, A. C., S. S. Nor’e, J. M. McCaw, H. R. Fryer, J. Mosse, A. R. McLean, and I. G. Barr. 2010. Assessing the viral fitness of oseltamivir-resistant influenza viruses in ferrets, using a competitive-mixtures model. Journal of virology 84:9427–38.

Jackson, S., N. Van Hoeven, L.-M. Chen, T. R. Maines, N. J. Cox, J. M. Katz, and R. O. Donis. 2009. Reassortment between avian H5N1 and human H3N2 influenza viruses in ferrets: a public health risk assessment. Journal of virology 83:8131–40.

Karlsson, E. A., V. A. Meliopoulos, C. Savage, B. Livingston, A. Mehle, and S. Schultz-Cherry. 2015. Visualizing real-time influenza virus infection, transmission and protection in ferrets. Nature communications 6:6378.

Langlois, R. A., R. A. Albrecht, B. Kimble, T. Sutton, J. S. Shapiro, C. Finch, M. Angel, M. A. Chua, A. S. Gonzalez-Reiche, K. Xu, D. Perez, A. García-Sastre, and B. R. tenOever. 2013. MicroRNA-based strategy to mitigate the risk of gain-of-function influenza studies. Nature biotechnology 31:844–7.

Lee, Y.-N., D.-H. Lee, J.-K. Park, S.-S. Yuk, J.-H. Kwon, S.-S. Nahm, J.-B. Lee, S.-Y. Park, I.-S. Choi, and C.-S. Song. 2013. Experimental infection and natural contact exposure of ferrets with canine influenza virus (H3N2). The Journal of general virology 94:293–7.

Maines, T. R., L.-M. Chen, N. Van Hoeven, T. M. Tumpey, O. Blixt, J. A. Belser, K. M. Gustin, M. B. Pearce, C. Pappas, J. Stevens, N. J. Cox, J. C. Paulson, R. Raman, R. Sasisekharan, J. M. Katz, and R. O. Donis. 2011. Effect of receptor binding domain mutations on receptor binding and transmissibility of avian influenza H5N1 viruses. Virology 413:139–47.

Maines, T. R., L.-M. Chen, Y. Matsuoka, H. Chen, T. Rowe, J. Ortin, A. Falcón, T. H. Nguyen, L. Q. Mai, E. R. Sedyaningsih, S. Harun, T. M. Tumpey, R. O. Donis, N. J. Cox, K. Subbarao, and J. M. Katz. 2006. Lack of transmission of H5N1 avian-human reassortant influenza viruses in a ferret model. Proceedings of the National Academy of Sciences of the United States of America 103:12121–6.

Maines, T. R., A. Jayaraman, J. A. Belser, D. A. Wadford, C. Pappas, H. Zeng, K. M. Gustin, M. B. Pearce, K. Viswanathan, Z. H. Shriver, R. Raman, N. J. Cox, R. Sasisekharan, J. M. Katz, and T. M. Tumpey. 2009. Transmission and pathogenesis of swine-origin 2009 A(H1N1) influenza viruses in ferrets and mice. Science (New York, N.Y.) 325:484–7.

Memoli, M. J., A. S. Davis, K. Proudfoot, D. S. Chertow, R. J. Hrabal, T. Bristol, and J. K. Taubenberger. 2011. Multidrug-resistant 2009 pandemic influenza A(H1N1) viruses maintain fitness and transmissibility in ferrets. The Journal of infectious diseases 203:348–57.

Memoli, M. J., R. J. Hrabal, A. Hassantoufighi, B. W. Jagger, Z.-M. Sheng, M. C. Eichelberger, and J. K. Taubenberger. 2010. Rapid selection of a transmissible multidrug-resistant influenza A/H3N2 virus in an immunocompromised host. The Journal of infectious diseases 201:1397–403.

Oh, D. Y., S. Lowther, J. M. McCaw, S. G. Sullivan, S.-K. Leang, J. Haining, R. Arkinstall, A. Kelso, J. Mcvernon, I. G. Barr, D. Middleton, and A. C. Hurt. 2014. Evaluation of oseltamivir prophylaxis regimens for reducing influenza virus infection, transmission and disease severity in a ferret model of household contact. The Journal of antimicrobial chemotherapy 69:2458–69.

Oxford, J. S., R. Lambkin, M. Guralnik, R. A. Rosenbloom, M. P. Petteruti, K. Digian, and C. LeFante. 2007. In vivo prophylactic activity of QR-435 against H3N2 influenza virus infection. American journal of therapeutics 14:462–8.

Pappas, C., K. Viswanathan, A. Chandrasekaran, R. Raman, J. M. Katz, R. Sasisekharan, and T. M. Tumpey. 2010. Receptor Specificity and Transmission of H2N2 Subtype Viruses Isolated from the Pandemic of 1957. PLoS ONE 5:e11158.

Pappas, C., H. Yang, P. J. Carney, M. B. Pearce, J. M. Katz, J. Stevens, and T. M. Tumpey. 2015. Assessment of transmission, pathogenesis and adaptation of H2 subtype influenza viruses in ferrets. Virology 477:61–71.

Pizzorno, A., Y. Abed, X. Bouhy, E. Beaulieu, C. Mallett, R. Russell, and G. Boivin. 2012. Impact of mutations at residue I223 of the neuraminidase protein on the resistance profile, replication level, and virulence of the 2009 pandemic influenza virus. Antimicrobial agents and chemotherapy 56:1208–14.

Roberts, K. L., H. Shelton, M. Scull, R. Pickles, and W. S. Barclay. 2011. Lack of transmission of a human influenza virus with avian receptor specificity between ferrets is not due to decreased virus shedding but rather a lower infectivity in vivo. The Journal of general virology 92:1822–31.

Roberts, K. L., H. Shelton, P. Stilwell, and W. S. Barclay. 2012. Transmission of a 2009 H1N1 pandemic influenza virus occurs before fever is detected, in the ferret model. PloS one 7:e43303.

Seibert, C. W., M. Kaminski, J. Philipp, D. Rubbenstroth, R. a Albrecht, F. Schwalm, S. Stertz, R. a Medina, G. Kochs, A. García-Sastre, P. Staeheli, and P. Palese. 2010. Oseltamivir-resistant variants of the 2009 pandemic H1N1 influenza A virus are not attenuated in the guinea pig and ferret transmission models. Journal of virology 84:11219–26.

The SJCEIRS Working Group. 2013. Assessing the fitness of distinct clades of influenza A (H9N2) viruses. Emerging Microbes & Infections 2:e75.

Wan, H., E. M. Sorrell, H. Song, M. J. Hossain, G. Ramirez-Nieto, I. Monne, J. Stevens, G. Cattoli, I. Capua, L.-M. Chen, R. O. Donis, J. Busch, J. C. Paulson, C. Brockwell, R. Webby, J. Blanco, M. Q. Al-Natour, and D. R. Perez. 2008. Replication and transmission of H9N2 influenza viruses in ferrets: evaluation of pandemic potential. PloS one 3:e2923.

Wong, D. D. Y., K.-T. Choy, R. W. Y. Chan, S. F. Sia, H.-P. Chiu, P. P. H. Cheung, M. C. W. Chan, J. S. M. Peiris, and H.-L. Yen. 2012. Comparable fitness and transmissibility between oseltamivir-resistant pandemic 2009 and seasonal H1N1 influenza viruses with the H275Y neuraminidase mutation. Journal of virology 86:10558–70.

Yen, H.-L., L. M. Herlocher, E. Hoffmann, M. N. Matrosovich, A. S. Monto, R. G. Webster, and E. A. Govorkova. 2005. Neuraminidase inhibitor-resistant influenza viruses may differ substantially in fitness and transmissibility. Antimicrobial agents and chemotherapy 49:4075–84.

Yen, H.-L., C.-H. Liang, C.-Y. Wu, H. L. Forrest, A. Ferguson, K.-T. Choy, J. Jones, D. D.-Y. Wong, P. P.-H. Cheung, C.-H. Hsu, O. T. Li, K. M. Yuen, R. W. Y. Chan, L. L. M. Poon, M. C. W. Chan, J. M. Nicholls, S. Krauss, C.-H. Wong, Y. Guan, R. G. Webster, R. J. Webby, and M. Peiris. 2011. Hemagglutinin-neuraminidase balance confers respiratory-droplet transmissibility of the pandemic H1N1 influenza virus in ferrets. Proceedings of the National Academy of Sciences of the United States of America 108:14264–9.

Yen, H.-L., A. S. Lipatov, N. A. Ilyushina, E. A. Govorkova, J. Franks, N. Yilmaz, A. Douglas, A. Hay, S. Krauss, J. E. Rehg, E. Hoffmann, and R. G. Webster. 2007. Inefficient transmission of H5N1 influenza viruses in a ferret contact model. Journal of virology 81:6890–8.

Yen, H.-L., J. Zhou, K.-T. Choy, S. F. Sia, O. Teng, I. H. Ng, V. J. Fang, Y. Hu, W. Wang, B. J. Cowling, J. M. Nicholls, Y. Guan, and J. S. M. Peiris. 2014. The R292K Mutation That Confers Resistance to Neuraminidase Inhibitors Leads to Competitive Fitness Loss of A/Shanghai/1/2013 (H7N9) Influenza Virus in Ferrets. Journal of Infectious Diseases 210:1900–1908.

Zanin, M., B. Marathe, S.-S. Wong, S.-W. Yoon, E. Collin, C. Oshansky, J. Jones, B. Hause, and R. Webby. 2015. Pandemic Swine H1N1 Influenza Viruses with Almost Undetectable Neuraminidase Activity Are Not Transmitted via Aerosols in Ferrets and Are Inhibited by Human Mucus but Not Swine Mucus. Journal of Virology 89:5935–5948.

Zaraket, H., T. Baranovich, B. S. Kaplan, R. Carter, M.-S. Song, J. C. Paulson, J. E. Rehg, J. Bahl, J. C. Crumpton, J. Seiler, M. Edmonson, G. Wu, E. Karlsson, T. Fabrizio, H. Zhu, Y. Guan, M. Husain, S. Schultz-Cherry, S. Krauss, R. McBride, R. G. Webster, E. A. Govorkova, J. Zhang, C. J. Russell, and R. J. Webby. 2015. Mammalian adaptation of influenza A(H7N9) virus is limited by a narrow genetic bottleneck. Nature communications 6:6553.

Zaraket, H., O. A. Bridges, S. Duan, T. Baranovich, S.-W. Yoon, M. L. Reed, R. Salomon, R. J. Webby, R. G. Webster, and C. J. Russell. 2013. Increased acid stability of the hemagglutinin protein enhances H5N1 influenza virus growth in the upper respiratory tract but is insufficient for transmission in ferrets. Journal of virology 87:9911–22.

Zhu, H., D. Wang, D. J. Kelvin, L. Li, Z. Zheng, S.-W. Yoon, S.-S. Wong, A. Farooqui, J. Wang, D. Banner, R. Chen, R. Zheng, J. Zhou, Y. Zhang, W. Hong, W. Dong, Q. Cai, M. H. A. Roehrl, S. S. H. Huang, A. A. Kelvin, T. Yao, B. Zhou, X. Chen, G. M. Leung, L. L. M. Poon, R. G. Webster, R. J. Webby, J. S. M. Peiris, Y. Guan, and Y. Shu. 2013. Infectivity, transmission, and pathology of human-isolated H7N9 influenza virus in ferrets and pigs. Science (New York, N.Y.) 341:183–6.
