# Supplementary material for: Mapping influenza transmission in the ferret model to transmission in humans
Source: eLife. 2015 Sep 2;4:e07969. doi: 10.7554/eLife.07969 (PMC4586390; doi:10.7554/eLife.07969)
Supplement: Figure 1—source data 4. — DOI: http://dx.doi.org/10.7554/eLife.07969.007 [file elife07969s004.docx]

**Figure 1 – source data 4:** Ferret influenza transmission studies via respiratory droplets (RD) and direct contact (DC) using avian isolates. Transmission was determined using seroconversion and/or viral isolation.

| **Route** | **Subtype** | **Isolate** | **Successful transmissions** | **Ferrets exposed** | **Reference** |
| --- | --- | --- | --- | --- | --- |
| **RD** | **H5N1** | A/Mallard Duck/Korea/W401/2011 | **0** | **2** | (Kwon et al. 2011) |
|  |  | A/Wild Bird/Hong Kong/07035-1/2011 | **0** | **3** | (Xu et al. 2013) |
|  |  | A/Egret/Egypt/1162/2006 | **0** | **2** | (Chen et al. 2012) |
|  |  | A/Environment/Korea/W149/2006 | **0** | **2** | (Kwon et al. 2011) |
|  | **H7N9** | A/Chicken/Shanghai/S1053/2013 | **0** | **3** | (Zhang et al. 2013) |
|  |  | A/Pigeon/Shanghai/S1421/2013 | **1** | **3** | (Zhang et al. 2013) |
|  |  | A/Duck/Gunma/466/2011 | **0** | **3** | (Watanabe et al. 2013) |
| **DC** | **H5N1** | A/Egret/Egypt/1162/2006 | **0** | **2** | (Chen et al. 2012) |
|  | **H7N9** | A/Goose/Nebraska/17097-4/2011 | **0** | **3** | (Belser et al. 2013) |

**References**

Belser, J. A., C. T. Davis, A. Balish, L. E. Edwards, H. Zeng, T. R. Maines, K. M. Gustin, I. L. Martínez, R. Fasce, N. J. Cox, J. M. Katz, and T. M. Tumpey. 2013. Pathogenesis, transmissibility, and ocular tropism of a highly pathogenic avian influenza A (H7N3) virus associated with human conjunctivitis. Journal of virology 87:5746–54.

Chen, L.-M., O. Blixt, J. Stevens, A. S. Lipatov, C. T. Davis, B. E. Collins, N. J. Cox, J. C. Paulson, and R. O. Donis. 2012. In vitro evolution of H5N1 avian influenza virus toward human-type receptor specificity. Virology 422:105–13.

Kwon, H.-I., M.-S. Song, P. N. Q. Pascua, Y. H. Baek, J. H. Lee, S.-P. Hong, J.-B. Rho, J.-K. Kim, H. Poo, C.-J. Kim, and Y. K. Choi. 2011. Genetic characterization and pathogenicity assessment of highly pathogenic H5N1 avian influenza viruses isolated from migratory wild birds in 2011, South Korea. Virus research 160:305–15.

Watanabe, T., M. Kiso, S. Fukuyama, N. Nakajima, M. Imai, S. Yamada, S. Murakami, S. Yamayoshi, K. Iwatsuki-Horimoto, Y. Sakoda, E. Takashita, R. McBride, T. Noda, M. Hatta, H. Imai, D. Zhao, N. Kishida, M. Shirakura, R. P. de Vries, S. Shichinohe, M. Okamatsu, T. Tamura, Y. Tomita, N. Fujimoto, K. Goto, H. Katsura, E. Kawakami, I. Ishikawa, S. Watanabe, M. Ito, Y. Sakai-Tagawa, Y. Sugita, R. Uraki, R. Yamaji, A. J. Eisfeld, G. Zhong, S. Fan, J. Ping, E. A. Maher, A. Hanson, Y. Uchida, T. Saito, M. Ozawa, G. Neumann, H. Kida, T. Odagiri, J. C. Paulson, H. Hasegawa, M. Tashiro, and Y. Kawaoka. 2013. Characterization of H7N9 influenza A viruses isolated from humans. Nature 501:551–5.

Xu, L., L. Bao, J. Yuan, F. Li, Q. Lv, W. Deng, Y. Xu, Y. Yao, P. Yu, H. Chen, K.-Y. Yuen, and C. Qin. 2013. Antigenicity and transmissibility of a novel clade 2.3.2.1 avian influenza H5N1 virus. The Journal of general virology 94:2616–26.

Zhang, Q., J. Shi, G. Deng, J. Guo, X. Zeng, X. He, H. Kong, C. Gu, X. Li, J. Liu, G. Wang, Y. Chen, L. Liu, L. Liang, Y. Li, J. Fan, J. Wang, W. Li, L. Guan, Q. Li, H. Yang, P. Chen, L. Jiang, Y. Guan, X. Xin, Y. Jiang, G. Tian, X. Wang, C. Qiao, C. Li, Z. Bu, and H. Chen. 2013. H7N9 influenza viruses are transmissible in ferrets by respiratory droplet. Science (New York, N.Y.) 341:410–4.
