# Supplementary material for: Mapping influenza transmission in the ferret model to transmission in humans
Source: eLife. 2015 Sep 2;4:e07969. doi: 10.7554/eLife.07969 (PMC4586390; doi:10.7554/eLife.07969)
Supplement: Figure 4—source data 1. — DOI: http://dx.doi.org/10.7554/eLife.07969.017 [file elife07969s005.docx]

**Figure 4 – source data 1:** Ferret influenza transmission studies via respiratory droplets using strains from gain-of-function experiments with H5N1 avian influenza and the reconstructed 1918 pandemic H1N1 strain. Transmission was determined using seroconversion (SC) and/or viral isolation (VI).

| **Subtype** | **Virus** | **Successful transmissions – SC/VI (VI only)** | **Ferrets exposed** | **Reference** |
| --- | --- | --- | --- | --- |
| **GOF H5N1** | HA(N158D/N224K/Q226L)/CA04 | **5(2)** | **6** | (Imai et al. 2012) |
|  | HA(N158D/N224K/Q226L/T318I)/CA04 | **6(4)** | **6** | (Imai et al. 2012) |
|  | HA(Q222L/G224S) PB2(E627K) – Passage 10 | **3** | **4** | (Herfst et al. 2012) |
| **1918 H1N1** | Unknown | **2** | **3** | (Imai et al. 2012) |
|  | A/South Carolina/1/18 | **3** | **3** | (Tumpey et al. 2007) |

**References**

Herfst, S., E. J. A. Schrauwen, M. Linster, S. Chutinimitkul, E. de Wit, V. J. Munster, E. M. Sorrell, T. M. Bestebroer, D. F. Burke, D. J. Smith, G. F. Rimmelzwaan, A. D. M. E. Osterhaus, and R. A. M. Fouchier. 2012. Airborne transmission of influenza A/H5N1 virus between ferrets. Science (New York, N.Y.) 336:1534–41.

Imai, M., T. Watanabe, M. Hatta, S. C. Das, M. Ozawa, K. Shinya, G. Zhong, A. Hanson, H. Katsura, S. Watanabe, C. Li, E. Kawakami, S. Yamada, M. Kiso, Y. Suzuki, E. A. Maher, G. Neumann, and Y. Kawaoka. 2012. Experimental adaptation of an influenza H5 HA confers respiratory droplet transmission to a reassortant H5 HA/H1N1 virus in ferrets. Nature 486:420–8.

Tumpey, T. M., T. R. Maines, N. Van Hoeven, L. Glaser, A. Solórzano, C. Pappas, N. J. Cox, D. E. Swayne, P. Palese, J. M. Katz, and A. García-Sastre. 2007. A two-amino acid change in the hemagglutinin of the 1918 influenza virus abolishes transmission. Science 315:655–9.
